# Comprehensive assessment of physical activity policies and initiatives in Saudi Arabia 2016–2022

**DOI:** 10.3389/fpubh.2023.1236287

**Published:** 2023-07-19

**Authors:** Mezna A. AlMarzooqi, Reem F. Alsukait, Ghadeer S. Aljuraiban, Shaima A. Alothman, Reem AlAhmed, Severin Rakic, Christopher H. Herbst, Hazzaa M. Al-Hazzaa, Saleh A. Alqahtani

**Affiliations:** ^1^Leaders Development Institute, Ministry of Sport, Riyadh, Saudi Arabia; ^2^Department of Community Health Sciences, College of Applied Medical Sciences, King Saud University, Riyadh, Saudi Arabia; ^3^World Bank Group, Washington, DC, United States; ^4^Lifestyle and Health Research Center, Health Sciences Research Center, Princess Nourah Bint Abdulrahman University, Riyadh, Saudi Arabia; ^5^Liver Transplant Center, King Faisal Specialist Hospital and Research Center, Riyadh, Saudi Arabia; ^6^Division of Gastroenterology and Hepatology, Johns Hopkins University, Baltimore, MD, United States

**Keywords:** physical activity, policy, physical activity initiative, promotion, Saudi Arabia

## Abstract

**Objective:**

This study aimed to review health-enhancing physical activity (HEPA) policies and initiatives introduced in Saudi Arabia (SA) since 2016 and identify the gaps in their design and implementation.

**Methods:**

A combination of methods was used, including semi-structured interviews with key informants from relevant entities (such as those from the ministries of health, education, sports, tourism, and other regulatory bodies) and a review of policy/initiative documents provided by them. Stakeholder mapping led by local experts and snowball sampling supported the identification of key informants. Three existing frameworks—the World Health Organization’s HEPA Policy Audit Tool, the Global Observatory for Physical Activity (PA) Policy Inventory, and the European Monitoring Framework for PA Indicators—were used to develop data collection instruments.

**Results:**

The review identified 44 policies/initiatives from different sectors. The Saudi Sports for All Federation is the leader in PA promotion and community sports development. However, there is a lack of multisectoral agenda and governance structures for PA promotion. The overlap between initiatives by different key informants results in duplication of efforts, including initiatives to promote PA among the general public led by competitive professional sports and community-based sports.

**Conclusion:**

The study findings indicate that several policies/initiatives have been implemented in SA since 2016. However, there is a need to focus on the challenges or barriers that affect the sustainability of policies/initiatives. A system-based approach can help build on sectoral synergies, thereby accelerating progress in engaging the Saudi population with PA.

## 1. Introduction

Enormous economic developments in Saudi Arabia (SA) in recent decades have been accompanied by modernization, lifestyle transformation, rapid demographic changes, and extensive urbanization. Despite the economic progress, SA, as many other countries with high economic standard, faces the threat of an increase in the prevalence of lifestyle-related diseases (1). Sedentary lifestyle is associated with increased risk non-communicable diseases and premature death (2). Concerted efforts are required to address the numerous barriers to physical activity (PA), such as road traffic issues; poor air quality; financial barriers; and a lack of infrastructure, time, and motivation to exercise (3–5). Promoting PA and helping to overcome obstacles are essential determinants of PA levels (6). Furthermore, nearly 90% of people globally live in countries that could significantly improve their PA promotion capacity, including possible improvements in their PA policies (7).

SA has acknowledged these challenges and developed policies and initiatives to promote health-enhancing PA (HEPA). In 2016, the Saudi Vision 2030, a framework to diversify the economy and strengthen various sectors, such as health, education, recreation, and tourism, was launched (8). Incorporated into this framework is the objective of creating a vibrant society, which includes a focus on healthy lifestyles. Some previous initiatives to promote PA in SA were implemented by agencies in different sectors—such as health, education, sports, environment, transportation, tourism, and urban design—either alone or in collaboration (4).

The Quality-of-Life Program, launched in 2018, has supported the realization of Saudi Vision 2030 by implementing initiatives to improve quality of life, in part by enhancing participation in sports and athletic activities (9). This has contributed to the development and establishment of the Ministry of Sport and the Sports For All Federation (SFA), which aim to increase sports and PA levels in SA (10). Monitoring policies is an integral step in gaging success and facilitating reforms. A review exploring the published scientific literature on the governments’ HEPA policies called for more research to assess implementation, sustainability, and outcomes (11).

Local evidence on sport and physical activity policies is limited (12, 13). For instance, semi-structured interviews conducted with 25 public health leaders, national program directors, and program implementation staff from the Ministry of Health indicated that effective national non-communicable diseases (NCDs) policies and strategies have a critical role when controlling chronic disease epidemics. The interviewees stated that faster development and implementation were achieved for policies on tobacco, sugar-sweetened drinks and obesity, compared with PA policies (12). Also, a recent analysis of sports and physical activity promotion initiatives and policies in Saudi Arabia, using semi-structured interviews conducted with stakeholders revealed that the most obvious achievement has been the rapid cultural change enabling women’s sports and promoting physical activity among Saudis (13).

There are several existing frameworks for analyzing PA policies and initiatives. Three such frameworks are the World Health Organization’s (WHO) HEPA Policy Audit Tool (PAT) (14), the Global Observatory for PA Policy Inventory Tool (15, 16), and the European Union Monitoring Framework for PA Indicators (EUMF) (16). The WHO HEPA PAT has demonstrated its ability to enhance understanding and inform PA policies (17). Some studies suggested that it would be beneficial to use the WHO HEPA PAT in conjunction with the EUMF because the former includes different research elements and the option to consult experts for their opinions (14, 17). These frameworks are recognized and used internationally, facilitating comparisons between countries and regions (14, 17).

A comprehensive overview of the current and planned PA-related policies and initiatives aimed at achieving the Saudi Vision 2030 targets can improve the policymaking processes and policy decisions (18). The primary objectives of this study are to review the current and planned HEPA policies that promote PA and reduce sedentary behavior (SB) and to identify the gaps in their design and implementation in SA.

## 2. Methods

For the review, a policy was defined as a written document, usually issued by an organization, that clearly states priorities, aims, and objectives, while an initiative is a specific intervention or plan in various settings (17). Relevant PA and SB policies and initiatives issued or launched in SA between 2016 and 2022 were included. Competitive sports committees (e.g., the Saudi Olympics Committee) were excluded because the study focused on PA and SB policies and initiatives in community settings. A combination of methods was used to identify the relevant PA and SB policies and initiatives. This included a review of documents—identified through the administration of the pre-interview data collection tool or online resources at key stakeholders’ websites–and semi-structured interviews with the key informants. Stakeholder mapping, conducted with the support of local experts, supported the identification of potential informants in key government institutions—ministries of health, education, sports, urban design, and tourism—and other regulatory bodies, whom we invited to participate in the study. Additional key informants were identified through snowball sampling. The key informants were initially contacted by email or telephone to confirm their willingness to participate in the study. Those who agreed to participate in the study were asked to sign an informed consent form. The Institutional Ethics Committee of King Saud University reviewed and approved the study protocol (21/01216/IRB).

### 2.1. Pre-interview data collection tool

The key informants who agreed to participate in the study were invited to complete the data collection tool prior to the interview. The tool was mainly based on the WHO HEPA PAT (version 2.0) (19). It was modified to include PA and SB policies and initiatives in SA (local and subnational), and included definitions of the key terms, to avoid misinterpreting the questions. Additionally, the Global Observatory for PA Policy Inventory Tool (15) and the EUMF (16) were consolidated for other required data, for example, data on implementation of policies (see Supplementary data).

The tool—a self-administered survey—required the participants to list the details of each policy/initiative they were familiar with, including its title, description, publication year, and issuing body. Participants were requested to grade the extent to which each policy/initiative was implemented. If a policy/initiative had been fully implemented, it was given a grade of 10. If most of the policy/initiative had been implemented, it was graded between 7 and 9; if it had been partially implemented, it was graded between 4 and 6. If policy statements/initiatives had been only minimally implemented, it was graded from 1 to 3; if none had been implemented, it was graded 0. Finally, participants were asked if there were quantifiable targets, key performance indicators, or an evaluation component for these policies/initiatives.

### 2.2. Key informant interviews

Qualitative data were collected through semi-structured interviews conducted by the authors (GA, RA, and MA) between October 2021 and February 2022. All authors had previous experience in interviewing. The interview guide and informed consent form in English were developed specifically for the current study (see Supplementary data). Three existing frameworks guided the formulation of the interview questions (14–16, 19). Two researchers independently reviewed the frameworks, selected the most relevant questions, and consolidated them into a single interview guide. The semi-structured interviews with key informants focused on their institutions’ role and leadership regarding PA promotion or SB reduction. The guide and pre-interview data collection tool were pilot-tested and further refined with the help of local experts. The interviews were conducted in Arabic or English, via either online meetings or telephone calls. The average length of the interview was 50 min. All key informants were asked questions directly from the interview guide in the same sequence, but the interviewers probed inductively to gain further information on achieving goals and targets, monitoring systems, communication strategies, and the challenges and opportunities faced by different sectors in promoting PA in SA. Detailed notes were taken to avoid the risk that recording would reduce the key informants’ willingness to disclose their attitudes. The notes from the interviews conducted in Arabic were translated into English.

Two researchers (GA and RA) simultaneously conducted qualitative content analysis to interpret meaning from the content of text data (20). Relevant text was coded/sorted into themes using inductive coding, where codes were developed as data were analyzed. Notes were then pooled, transcribed collaboratively by team members, and were compared to improve interpretation.

## 3. Results

**Figure 1:**
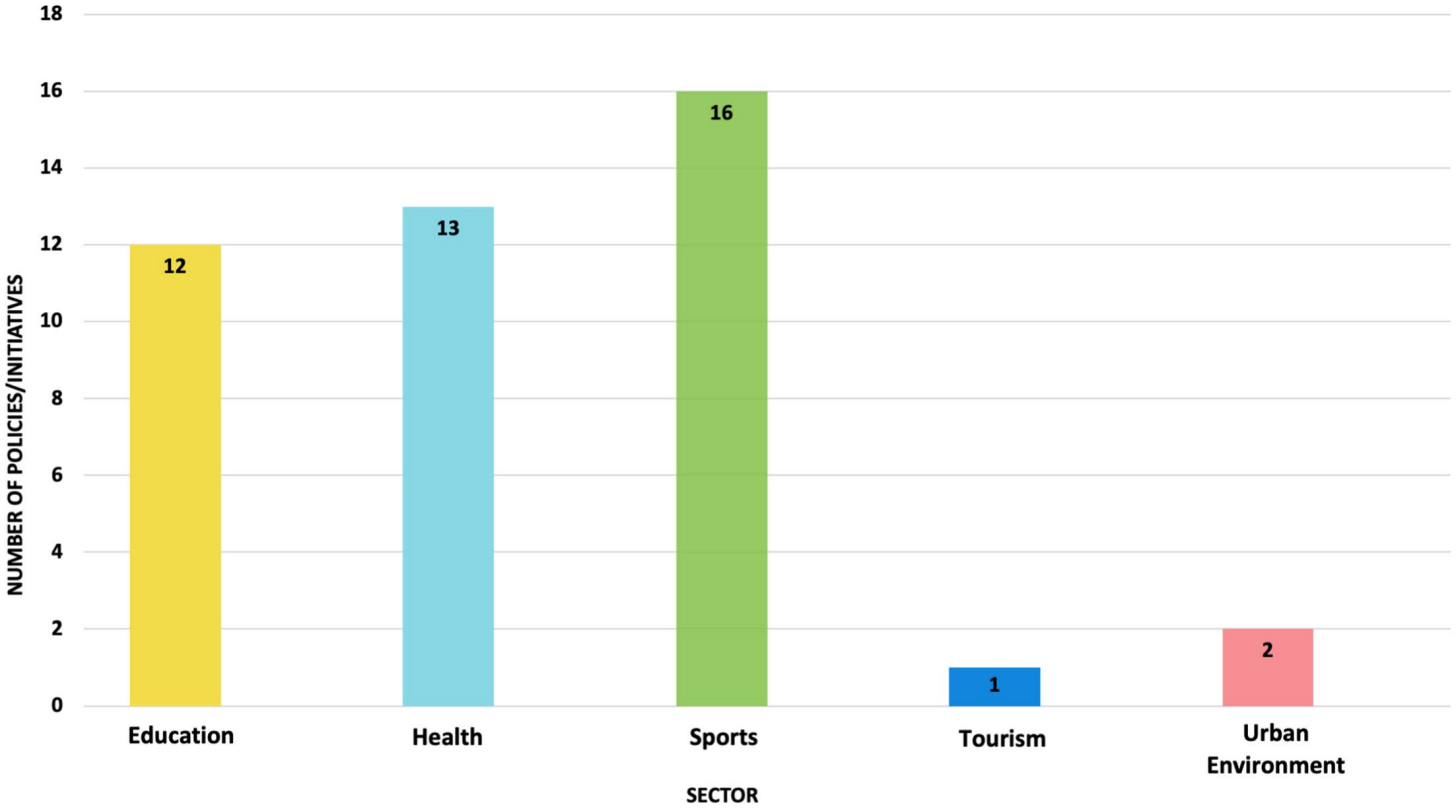
Number of physical activity and sedentary behaviors policies/initiatives by sector.

Of the 15 key informants invited, 10 participated in the study. The participants were from the following sectors: health, urban environment, tourism, education, and sports. Three of the 10 participants filled out the pre-interview data collection tool, while the remaining seven provided the required information during the interview. The document review process resulted in the identification of 25 documents detailing 44 policies/initiatives from the following sectors: health (*n* = 13), education (*n* = 12), sports (*n* = 16), tourism (*n* = 1), and urban environment (*n* = 2) (Figure 1).

### 3.1. Key informants involved in PA promotion and SB reduction

Multiple key informants were actively involved in PA promotion and SB reduction in SA. Their roles are described in Table 1. The mandate for overall coordination and leadership on these issues remained unclear. Some of the key informants identified the Public Health Authority, the Ministry of Health, and the SFA as the leaders for community sports and PA promotion. The SFA was also reported to be a leader in coordinating the implementation of PA promotion across different sectors.

**Table 1:** Examples of key stakeholders in the promotion of physical activity in Saudi Arabia.

Sector	Stakeholder	Role
Health	Health in All Policies	A ministerial committee was formed in 2017 Led by the Ministry of Health Included memberships from the Ministry of Education, Ministry of Commerce, Ministry of Municipal and Rural Affairs and Housing, Ministry of Labor and Social Development, and Ministry of Environment, Water, and Agriculture One of its priority areas is to support healthy lifestyles among the general public
	Ministry of Health: Health Programs	Develops programs that promote health and prevent chronic diseases for which physical inactivity is an identified risk factor
	Ministry of Health: Healthy Cities	The WHO Healthy Cities initiative was conceived to place health high on the social and political agenda of cities by promoting health, equity, and sustainable development through innovation and multisectoral change
	Public Health Authority	Aims to protect and promote public health and prevent disease
	Public Health at the Gulf Health Council	Promotes and enhances the health sector of all member states by providing proactive initiatives and responding to regional and global health challenges Increases health awareness among the citizens of these states through special programs to promote physical activity (PA)
Education	Ministry of Education: School Health	Promotes the health and physical activity behavior of the school community by raising awareness and developing programs to increase PA
	Ministry of Education: Saudi Schools Sports Federation	Encourages a strong sports culture among all students Raises community awareness of PA Promotes school sports in all aspects through trainings and hosting tournaments
	Ministry of Education: Saudi Universities Sports Federation	Supports and develops Saudi universities’ sports sector Incorporates PA into the curricula
	Nourah Health Program, Princess Nourah Bint Abdulrahman University (Riyadh City, Central Region)	First Saudi university to receive WHO accreditation for the Healthy University Program as part of the Healthy City Program Promotes PA through numerous policies, initiatives, and facilities, particularly for women
Sports	Ministry of Sport	Regulates the sports sector, promotes its components, and provides modern facilities to broaden the base of sports practitioners and achieve regional and global excellence Its vision is to achieve an athletic environment and a distinguished competitive sports field
	Sports for All Federation	Established in 2018 under the Ministry of Sport The main body responsible for developing community sports Mandated to achieve a target of raising physical activity levels to at least once a week among at least 40% of the population by 2030
Tourism	Saudi Tourism Authority	Develops and promotes national leisure packages that frequently include PA (e.g., hiking, diving, climbing, and other outdoor adventures) for both domestic and international tourists
Urban environment	Ministry of Municipal and Rural Affairs and Housing	Ensures public spaces are optimized for PA Part of the Healthy Cities Activation Committee in collaboration with the Ministry of Health Riyadh Region Municipality under the Ministry of Municipal and Rural Affairs partnered with the Gulf Health Council on activating QR codes with promotional messages at King Abdullah Walkway, Riyadh Indirectly promoting sports and physical activity by encouraging the building of new housing compounds with sports facilities that cater to the local context
	Royal Commission of Riyadh City Development Authority–Health Department	Newly established as part of the healthcare department Fosters a healthy lifestyle by promoting physical and mental wellness Promotes quality of life objectives of the Saudi Vision 2030
	Royal Commission of Riyadh City Development Authority–Sports Boulevard	Encourages citizens of Riyadh to follow a healthy lifestyle through exercise and participation in various sporting activities Provides safe, green pathways for pedestrians, special routes for professional and amateur cyclists, horseback riding paths, and designated sites for sporting activities	
	NEOM Project	New urban model for sustainable living, working, and prospering, with facilities to promote PA Provides unique development opportunities in the coastal Red Sea, including various sporting activities and green pathways for pedestrians
Civil society	Kayl Association for Combating Obesity	Founded in 2010 One of their programs is PA options (other than gyms) The association is based in Riyadh but carries out work throughout Saudi Arabia (SA) to pursue nationwide healthcare and obesity goals as part of the Saudi Vision 2030
Others	Quality of Life program	Launched in 2018 to improve the quality of life of residents and visitors in SA by developing the necessary environment to create more vibrant options that enhance the participation and experience of citizens and residents Includes promoting sports activities in the community Provides funds and monitors relevant PA promotion indicators
	National Center for Performance Measurement “Adaa”	An independent government body founded in 2016 publishes quarterly reports on the delivery progress of strategic goals, initiatives, and key performance indicators of public entities to track their development in realizing Saudi Vision 2030
	General Authority for Statistics	The official statistical reference for statistical data and information in SA Collects national-level surveys such as the sports survey and family health survey, which include PA indicators

PA, physical activity; QR, quick response; SA, Saudi Arabia; WHO, World Health Organization.

### 3.2. Political commitment toward PA promotion, policies, and initiatives

Many entities promoting PA in SA have recently been established as part of the Saudi Vision 2030 commitment to increasing PA level to at least once a week among at least 40% of the population by 2030 (9). Most objectives described in the 44 policies and initiatives referred to the overall Saudi Vision 2030 goal, with differences depending on their sector, target audience, and settings. Some examples of such policy documents include “National Diet and Physical Activity Strategy 2015–2025,” “Twenty-Four-Hour Movement Practice Guidelines for Saudi Arabia,” and “Provide PA Guideline for Health Practitioners” (21–23). There was a wide range of initiatives, such as the “Walk 30” national campaign by the Ministry of Health and the “Expat Youth Swimming Program” targeting expatriate youth in Riyadh city. Supplementary Table S1 provides further details on the policies/initiatives for each sector.

### 3.3. Monitoring and evaluation of PA promotion policies and initiatives

The key informants reported the use of several monitoring and evaluation frameworks. The Ministry of Health’s key informants reported using the 2019 World Health Survey (24). Other national-level surveys included the General Authority of Statistics’ Family Health Survey and Sports Survey (25, 26). The SFA reported using the Nielsen Global Health and Wellness Survey yearly to identify PA prevalence and preferences stratified by demographic categories. Other entities collected their own primary data, including process evaluation measures (e.g., number of participants) and baseline self-reported assessments of their target audiences. Of all the policies/initiatives reviewed, 30 were reported to have an evaluation component (Supplementary Table S1).

### 3.4. Strengths, challenges, and future plans

Interviewees reported that there was sufficient momentum to support PA promotion, especially from the leadership of their respective entities. The availability of key policy documents based on scientific evidence—such as those issued by the WHO and the United States Centers for Disease Control and Prevention–was a reported strength in all policies/initiatives. However, translating the evidence into implementation plans, cross-sectoral coordination to avoid duplication, and overall governance were recognized as the main challenges. For example, one key informant reported that “there is a need for clear guidance on HEPA policies, especially when it comes to defining, monitoring, and managing the national PA agenda.” Another challenge reported by almost all key informants was the limited engagement of the private sector, for example, through worksite wellness programs that promote PA. While some interviewees reported device-based measurement indicators as a challenge, others highlighted potential opportunities for utilizing global positioning systems (GPS) and mobile applications, such as the Sehhaty, to collect data. A summary of the strengths, challenges, and progress achieved regarding PA and SB policies/initiatives is provided in Table 2.

**Table 2:** Strengths, challenges, and progress achieved in promoting physical activity in Saudi Arabia, by sector.

Sector	Strengths	Challenges	Progress
Health	Presence of a local network of stakeholders Key policy documents based on the available international evidence	Absence of an evaluation and monitoring component in most initiatives Non-standardized measurement tools that may lead to inconsistent data reporting Suspension of several initiatives due to COVID-19 A lack of explicitly defined governance structure for PA promotion Limited funds specifically allocated to promoting PA Limited intersectoral coordination on PA promotion (despite a network of stakeholders), leading to a lack of alignment Restricted reach and partnerships with the community Challenges in involving communities and spreading awareness	Inclusion of a monitoring and evaluation component in every future policy (planning phase) Forthcoming implementation of a Vision Realization Program to enable Public Health Authority to collect data and set policies for PA in all sectors Initiation of several policies to cater to everyone in SA Mass awareness programs are being planned to reach individuals in remote areas, especially women. PA is promoted through the health sector to stimulate other sectors to improve access to PA facilities
Education	Important setting for the promotion of PA among young people (school and university students) Presence of a local network of stakeholders Key policy documents based on credible international research	Absence of clear governance on PA promotion Unavailability of reliable/representative PA data for all relevant demographic groups Limited mass media engagement for promoting PA among the community Challenges in facilitating community involvement Limited funding for promoting PA Insufficient number of professional female coaches to train young athletes	Incorporation of PA into girls’ school curricula Close partnership, specifically focusing on sports participation, established with the Sports for All Federation Record increases in the number of schools and universities participating in a variety of national and international leagues and championships Evaluation and monitoring components present in most initiatives at the school and university levels Suitable swimming classes to be provided soon to all school children
Sports	Development of a focused 5-year strategy to drive community sports and increase PA Nationwide reach Local network of stakeholders Promotion of PA Key policies based on best global practices, e.g., WHO guidelines, and national-level evidence, e.g., SFA	A lack of explicitly defined governance structure and stakeholder management when it comes to PA and community sports groups Potential replication of initiatives and lack of alignment between sectors regarding promoting PA Limited PA facilities	Strong cooperation and support from stakeholders Many initiatives launched and implemented Reach expanded to all segments of the population (locals and expatriates), especially women KSA now hosts several sports activities, such as the formula race and car rally events Provision of convenient permits and licenses leading to massive increases in the number of female fitness centers Several shopping malls now offer space for walking/jogging
Tourism	Close collaboration with the Ministry of Tourism, Tourism Development Fund, and wider travel and tourism ecosystem to achieve targets All products and packages based on an in-depth, data-driven understanding of customer wants and needs by the Tourism Data and Insights Team	Limited investment in the tourism infrastructure (e.g., adventure-themed attractions, health and wellness resorts, and coastal development with a focus on marine activities), which could facilitate PA	Consistent recording and tracking of the uptake in customer responses, especially those based on PA
Urban environment	Close collaboration with the Sports for All Federation and the Public Health Authority Key policies based on best global practices, e.g., WHO guidelines, and national-level evidence, e.g., SFA	A lack of explicitly defined governance structure when it comes to PA promotion Lack of a centralized database or access to valid data sources Lack of clear communication and understanding across various sectors regarding PA Restricted reach and partnerships with the community	Monitoring and evaluation components are to be implemented soon for all policies

## 4. Discussion

This comprehensive review of the current and planned PA-related policies and initiatives in Saudi Arabia shows that there has been unprecedented progress in promoting PA among the Saudi population since the launch of the Saudi Vision 2030. The policies have high-level mandates, and several new entities have been established to promote PA. The review shows that several stakeholders actively promoted PA in SA and highlights several strengths and challenges in this process.

The review identified 44 policies/initiatives that cut across different sectors, such as health, education, sports, tourism, and urban design. Currently, the SFA is the main stakeholder responsible for developing sports and PA in the community, which has already reached its 2030 goal of increasing the percentage of individuals exercising at least once a week from 13% of the population to 40% by 2030 (27). The stakeholders that actively promote PA policies/initiatives may help to achieve the 2030 goals for PA levels, as well as the WHO PA recommended levels of 150 min of vigorous PA and/or 300 min of moderate/aerobic PA per week (28).

PA policies in SA have seen many improvements. The greatest strength found in the health sector was the presence of a local network of stakeholders and policies based on best global practices, e.g., WHO guidelines, and national-level evidence, e.g., SFA. This is similar to an Italian study, which reported the involvement of several representatives in sustainable healthcare activities, providing healthcare services and equipment, pharmaceuticals, biotechnology, and related life sciences (29). Meanwhile, in the education sector, the greatest strength was the setting for the promotion of PA among young people (school and university students). Several studies show that schools and universities are important venues for promoting PA (30–32). As a result, policymakers, planners, and principals of different schools and universities have implemented and expanded policies that integrate regular PA in the classes (30, 33, 34). School PA policies guide the implementation of PA programs that contribute to preventing public health challenges such as obesity (35). The development of a 5-year strategy by the Sports for All Federation to drive community sports and increase PA was found to be one of the strengths. A long-term intervention plan can help to assess actual and potential impacts to improve access to different opportunities for PA (36). In addition, a long-term intervention plan can help to coordinate efforts of various agencies (36). The notable strength in promoting PA in the tourism and urban environment sectors was the close collaboration of different agencies, such as the Ministry of Tourism, the Tourism Development Fund, SFA, and Public Health Authority, to achieve targets. The concept of collaboration between agencies and stakeholders in policymaking has been a practice for a long time (37). Cross-sectoral cooperation has considerable advantages. It may promote innovation through various agencies’ experiences and improve understanding of policy implementation (37).

The review also found that most PA policies/initiatives focused on mass participation, such as creating tournaments and competitions, encouraging the involvement of different schools and universities, and establishing sports groups and community bodies. SFA appears to be the primary stakeholder in PA promotion and community sports development (38). The findings are similar to those of a previous narrative review of PA initiatives in SA (4). In addition, several PA counseling activities were provided for students and various population groups in SA (39). The study results also show that sports facilities and infrastructure are being built to promote PA among the general public. These findings align with the Global Action Plan on PA 2018–2030 strategic objective of creating active environments (3, 40), that include building facilities where people can engage in regular PA and promoting equitable access to safe places and public spaces in cities, towns, suburbs, and rural communities (3). Improving facilities is often regarded as a fundamental action to promote PA (41).

Several challenges were identified. One of the main challenges was the lack of a clear multisectoral agenda and explicitly defined governance structure for PA promotion in SA. The overlaps between different stakeholders’ initiatives have resulted in occasional duplication of efforts, including initiatives to promote PA among the general public led by competitive professional sports and community-based sports. These observations are similar to the findings of a study in Ireland, which found challenges to establishing connections between policy action in promoting PA and national initiatives or no policy action that aligned with the provision programs (42). Furthermore, there is a need to focus on the challenges that affect the sustainability of policies/initiatives. For example, leadership is needed in implementing and enhancing PA policies, particularly in defining, monitoring, and managing the national PA agenda. The current study’s findings are consistent with those of Al-Hazzaa (43), who found similar barriers to PA policies in SA. Using a system-based approach, like that described in the WHO’s Global Action Plan on Physical Activity 2018–2030, can help build on sectoral synergies, thereby accelerating progress in engaging the population in PA in SA.

The identified policies/initiatives varied greatly in their targeted groups, settings, and approaches. The majority of interviewees reported using process evaluation indicators. Accordingly, the effectiveness of these initiatives/policies could have been assessed using devices, such as accelerometers or GPS tracking systems. While process evaluation indicators, such as the number of participants or the number of viewers, are an important component of monitoring and evaluation, the evidence shows that they do not necessarily translate into behavioral changes, such as increasing levels of PA among the Saudi population (44). In addition, various methods were used to promote PA, such as podcasts and videos. Furthermore, promoting PA through new or non-traditional activities may be especially appealing to some population groups (45). For example, ice-skating could be promoted among older adults in Saudi Arabia, as this type of PA seems to be popular in this age group in some other countries, such as the UK (45). The findings were also similar to a study in China in which PA policies targeted different range of sectors such as children, adolescents and general population (46).

The present study has three key strengths: (1) the involvement of stakeholders in PA promotion as key informers; (2) covering a wide range of policies from different sectors; (3) the use of standardized frameworks for PA policy assessment. There are limitations that should be considered in this review. There was a small number of included policies, initiatives and key informants, particularly from the education sector. With the limited number of studies that reported each indicator, our findings may not be generalizable to all stakeholders in PA promotion and all PA policies. Hence, there is a need for larger studies that will use these indicators to analyze PA policies. Furthermore, not all elements of the Comprehensive Analysis of Policy on Physical Activity (CAPPA) framework were analysed in this study. Future research may need to examine the extent of PA policies in Saudi Arabia that covered each of the element of CAPPA framework.

This article has presented an overview of the available evidence on the current and planned HEPA policies introduced in SA since 2016 and the gaps in their design and implementation. One of the strengths regarding PA policies was that multiple stakeholders were actively involved in PA promotion and SB reduction in SA. In conclusion, the study shows that there were several policies/initiatives from different sectors have been implemented in SA since 2016. However, it is notable that there is a lack of multisectoral agenda and governance structures for PA promotion. The overlap between initiatives by different key informants results in duplication of efforts, highlighting the need to approach and focus on the challenges or barriers that affect the sustainability of policies/initiatives. A system-based approach can help build on sectoral synergies, thereby accelerating progress in engaging the Saudi population with PA. Results from this review also point to several challenges, such as the need for more explicit governance on PA promotion. The findings of this study may serve as inspiration and source of information for policymakers and stakeholders who intend to address these gaps. Stakeholders and participating agencies should tackle policy/initiatives challenges to facilitate the development and implementation of effective PA and SB policies.

## Data availability statement

The datasets presented in this article are not readily available because the datasets supporting the conclusions of this article are available at the King Saud University from the corresponding author on reasonable request. Requests to access the datasets should be directed to malmarzooqi@ksu.edu.sa.

## Ethics statement

The studies involving human participants were reviewed and approved by the Institutional Ethics Committee of King Saud University reviewed and approved the study protocol (21/01216/IRB). The patients/participants provided their written informed consent to participate in this study.

## Author contributions

MA, GA, SAA, RFA, RA, SR, CH, and HA-H: study concept. GA, RFA, and MA: data collection. RFA and GA: data analysis. MA and GA: drafting the paper. SA, CH, RA, SR, and HA-H: revising it critically for intellectual content. All authors contributed to the article and approved the submitted version.

## Funding

This work was supported by the King Faisal Specialist Hospital and Research Center and World Bank. Financing for the analysis was provided by the King Faisal Specialist Hospital and Research Center and the Health, Nutrition, and Population Reimbursable Advisory Services Programs between the World Bank and the Ministry of Finance in Saudi Arabia (P172148 and P179873). The content is solely the responsibility of the authors and does not necessarily represent the official views of the King Faisal Specialist Hospital and Research Center or the World Bank.

## Conflict of interest

The authors declare that the research was conducted in the absence of any commercial or financial relationships that could be construed as a potential conflict of interest.

## Publisher’s note

All claims expressed in this article are solely those of the authors and do not necessarily represent those of their affiliated organizations, or those of the publisher, the editors and the reviewers. Any product that may be evaluated in this article, or claim that may be made by its manufacturer, is not guaranteed or endorsed by the publisher.
