# Supplementary material for: Comprehensive assessment of physical activity policies and initiatives in Saudi Arabia 2016–2022
Source: Front Public Health. 2023 Jul 19;11:1236287. doi: 10.3389/fpubh.2023.1236287 (PMC10443594; doi:10.3389/fpubh.2023.1236287)
Supplement: Supplementary file 1 [file Data_Sheet_1.docx]

Supplementary Material

# Supplementary Data

## Annex 1. Pre-interview data collection tool:

| **DEPARTMENT NAME** |  |
| --- | --- |
| **COMPLETED BY (insert name)** |  |
| **DATE COMPLETED** |  |

**GLOSSARY**

| **Action plan** | An action plan should identify **who does what** (type of activities and people responsible for implementation), **when** (time frame), **how** (approaches, activities and interventions) and for **how much** (resources). It should ideally also have an inherent mechanism for monitoring and evaluation. An action plan can be part of a policy (see below) or be an independent document. |
| --- | --- |
| **(Health-enhancing) physical activity (HEPA)** | This is any form of physical activity (movement produced by skeletal muscles that results in energy expenditure) that benefits health and functional capacity, without undue harm or risk. |
| **Policy** | A policy is a written document that contains priorities, defines goals and objectives, and is usually issued by (part of) the public administration. It can contain or be accompanied by an action plan (see above).  Formal written law, codes, or regulations with legal authority;  Standards that guide choices (includes plans);  Social norms – unwritten rules that guide appropriate and inappropriate attitudes, beliefs, values, and behaviors |
| **Programme** | A programme is a set of measures or a single (but large-scale) long-term activity, which may or may not be related to a policy document. A programme can contain different types of activities, such as social marketing campaigns, promotional events, specific interventions or initiatives in different settings, and can be time limited or open ended. |
| **Strategy** | A strategy is defined by a long-term plan designed to achieve national goals (in this case, to promote health and prevent diseases). |
| **Surveillance / monitoring system** | A health surveillance/monitoring system is the continuous, systematic collection, analysis and interpretation of the health-related data needed for the planning, implementation, and evaluation of public health practice. |
| **Sedentary behaviour** | Sedentary behaviour is any waking behaviour involving very low energy expenditure in a sitting or reclining posture. Examples include watching television, reading, listening to music, etc. in a seated or reclining posture. Any behaviors done standing are not considered sedentary. |

**Section 1: Policy documents, legislation, strategies, or action plans (2016-2021):**

1. This section is about **any current policy documents legislation, strategies or action plans** directly or indirectly related to physical activity or reduction of sedentary behavior promotion agenda in your sector during the period between 2016-2021. Please list the documents and, if possible, provide a brief description of the general content of each document (about 100–250 words). Please repeat as needed. N/A = not applicable.

| **Document title (1):** | Don’t know | N/A |
| --- | --- | --- |
| Publication year: | Don’t know | N/A |
| Time frame covered: | Don’t know | N/A |
| Issuing body: | Don’t know | N/A |
| Includes an evaluation component: (yes/no) | Don’t know | N/A |
| Web link (please send a copy of the document by email to galjuraiban@ksu.edu.sa) | Don’t know | N/A |
| Brief description including the purpose of the document and how it impacts physical activity/sedentary behaviour (about 100-250 words): | | |
| **Document title (2):** | Don’t know | N/A |
| Publication year: | Don’t know | N/A |
| Time frame covered: | Don’t know | N/A |
| Issuing body: | Don’t know | N/A |
| Includes an evaluation component: (yes/no) | Don’t know | N/A |
| Web link (please send a copy of the document by email to galjuraiban@ksu.edu.sa) | Don’t know | N/A |
| Brief description including the purpose of the document and how it impacts physical activity/sedentary behaviour (about 100-250 words): | | |
| **Document title (3):** | Don’t know | N/A |
| Publication year: | Don’t know | N/A |
| Time frame covered: | Don’t know | N/A |
| Issuing body: | Don’t know | N/A |
| Includes an evaluation component: (yes/no) | Don’t know | N/A |
| Web link (please send a copy of the document by email to galjuraiban@ksu.edu.sa) | Don’t know | N/A |
| Brief description including the purpose of the document and how it impacts physical activity/sedentary behaviour (about 100-250 words): | | |
| **Document title (4):** | Don’t know | N/A |
| Publication year: | Don’t know | N/A |
| Time frame covered: | Don’t know | N/A |
| Issuing body: | Don’t know | N/A |
| Includes an evaluation component: (yes/no) | Don’t know | N/A |
| Web link (please send a copy of the document by email to galjuraiban@ksu.edu.sa) | Don’t know | N/A |
| Brief description including the purpose of the document and how it impacts physical activity/sedentary behaviour (about 100-250 words): | | |
| **Document title (5):** | Don’t know | N/A |
| Publication year: | Don’t know | N/A |
| Time frame covered: | Don’t know | N/A |
| Issuing body: | Don’t know | N/A |
| Includes an evaluation component: (yes/no) | Don’t know | N/A |
| Web link (please send a copy of the document by email to galjuraiban@ksu.edu.sa) | Don’t know | N/A |
| Brief description including the purpose of the document and how it impacts physical activity/sedentary behaviour (about 100-250 words): | | |

1. Please estimate to what extent have each of the above-mentioned programs, action plans, or strategies been **implemented.**
   - For example, a document may mention building new facilities as one of the strategies to increase participation in physical activity. Implementation of this statement means having the new facilities built.
   - If the specific program has been fully implemented, please grade its implementation as 10. Please grade the implementation of the program from 7 to 9, if most of its statements have been implemented. Please grade the implementation of a program from 4 to 6, if around a half of its statements have been implemented. Please grade the implementation of a program from 1 to 3, if only a minority of its statements have been implemented. If the program has not been implemented at all, please grade it as 0.

| **Document** | 0 | 1 | 2 | 3 | 4 | 5 | 6 | 7 | 8 | 9 | 10 | Don’t know | N/A |
| --- | --- | --- | --- | --- | --- | --- | --- | --- | --- | --- | --- | --- | --- |
| **“1”** |  |  |  |  |  |  |  |  |  |  |  |  |  |
| **“2”** |  |  |  |  |  |  |  |  |  |  |  |  |  |
| **“3”** |  |  |  |  |  |  |  |  |  |  |  |  |  |
| **“4”** |  |  |  |  |  |  |  |  |  |  |  |  |  |
| **“5”** |  |  |  |  |  |  |  |  |  |  |  |  |  |

1. Please provide the name of and/or link to the published sources (e.g. journal article, research document, technical report, thesis, dataset) that informed your answer above. Please provide this information for each of the above-mentioned items. Please write “my personal assessment” if your estimations were not informed by any other source.

| **Document** | **Implementation evidence source(s)** (title, publisher, date, weblink or my personal assessment) |
| --- | --- |
| **“1”** |  |
| **“2”** |  |
| **“3”** |  |
| **“4”** |  |
| **“5”** |  |
| **“6”** |  |

**Section 2: Goals, targets, and objectives and effectiveness:**

1. Do you have quantifiable targets or KPI’s related to physical activity or sedentary behavior (e.g. to increase the prevalence of meeting physical activity guidelines by 15% by 2030) for:

|  | Yes | No | Don’t know |
| --- | --- | --- | --- |
| Physical activity? |  |  |  |
| Sedentary behavior? |  |  |  |

1. If yes, provide information on your quantifiable goals or KPIs (these could be directly or indirectly linked to physical activity or sedentary behavior). Please specify if it it’s a population subgroup (e.g., children or elderly).

| 1. **Target:**   **Target group:** | | |
| --- | --- | --- |
| **Time frame (start-end):** | Don’t know | N/A |
| Provide the name of and/or link to the published sources (e.g. journal article, research document, technical report, thesis, dataset) that informed your answer above. Please provide this information for each of the above-mentioned items. Please write “my personal assessment” if your estimations were not informed by any other source.  **Effectiveness evidence source(s)** (title, publisher, date, weblink or my personal assessment): | | |
| **(2) Target:**  **Target group** | | |
| **Time frame (start-end):** | Don’t know | N/A |
| Provide the name of and/or link to the published sources (e.g. journal article, research document, technical report, thesis, dataset) that informed your answer above. Please provide this information for each of the above-mentioned items. Please write “my personal assessment” if your estimations were not informed by any other source.  **Effectiveness evidence source(s)** (title, publisher, date, weblink or my personal assessment): | | |
| **(3) Target:**  **Target group** | | |
| **Time frame (start-end):** | Don’t know | N/A |
| Provide the name of and/or link to the published sources (e.g. journal article, research document, technical report, thesis, dataset) that informed your answer above. Please provide this information for each of the above-mentioned items. Please write “my personal assessment” if your estimations were not informed by any other source.  **Effectiveness evidence source(s)** (title, publisher, date, weblink or my personal assessment): | | |
| **(4) Target:**  **Target group** | | |
| **Time frame (start-end):** | Don’t know | N/A |
| Provide the name of and/or link to the published sources (e.g. journal article, research document, technical report, thesis, dataset) that informed your answer above. Please provide this information for each of the above-mentioned items. Please write “my personal assessment” if your estimations were not informed by any other source.  **Effectiveness evidence source(s)** (title, publisher, date, weblink or my personal assessment): | | |
| **(5) Target:**  **Target group** | | |
| Time frame (start-end): | Don’t know | N/A |
| Provide the name of and/or link to the published sources (e.g. journal article, research document, technical report, thesis, dataset) that informed your answer above. Please provide this information for each of the above-mentioned items. Please write “my personal assessment” if your estimations were not informed by any other source.  **Effectiveness evidence source(s)** (title, publisher, date, weblink or my personal assessment): | | |

1. Please estimate to what extent have each of the above-mentioned programs, action plans, or strategies in the previous section been **effective**
   - Overall, it is considered effective, if, as a result of it, physical activity levels have increased (or remained stable) or sedentary behaviour has decreased (or remained stable) as planned.
     - If all targets have been met, please grade the effectiveness of y as 10. If most targets have been met, please grade the effectiveness from 7 to 9. If around half of the targets have been met, please grade the effectiveness from 4 to 6. If only a minority of the targets have been met, please grade the effectiveness from 1 to 3. If no targets have been met, please grade the effectiveness as 0.

| **Document** | 0 | 1 | 2 | 3 | 4 | 5 | 6 | 7 | 8 | 9 | 10 | Don’t know | N/A |
| --- | --- | --- | --- | --- | --- | --- | --- | --- | --- | --- | --- | --- | --- |
| **“1”** |  |  |  |  |  |  |  |  |  |  |  |  |  |
| **“2”** |  |  |  |  |  |  |  |  |  |  |  |  |  |
| **“3”** |  |  |  |  |  |  |  |  |  |  |  |  |  |
| **“4”** |  |  |  |  |  |  |  |  |  |  |  |  |  |
| **“5”** |  |  |  |  |  |  |  |  |  |  |  |  |  |

**Section 3: Settings, population groups, and international documents:**

1. Considering all the key physical activity policy documents listed above, please indicate which **settings** are included for the delivery of specific HEPA actions. Please only tick those settings in which dedicated programmes or interventions are foreseen or already under way.

| Preschools/kindergarten |  | Sport and recreation |  |
| --- | --- | --- | --- |
| Primary schools |  | Transport |  |
| Secondary/high schools |  | Tourism |  |
| Colleges/universities |  | Environment |  |
| Primary health care |  | Urban design and planning |  |
| Clinical health care (e.g. hospitals) |  | Community |  |
| Workplace |  | Other (please specify): | |
| Older adult/senior services |  |  |  |

1. Considering all the key physical activity policy documents listed above, please indicate which **population groups** are targeted by specific HEPA actions settings. Please only tick those settings for which dedicated programmes or interventions are foreseen or already under way.

| Early years |  | Sedentary/the least active |  |
| --- | --- | --- | --- |
| Children/young people |  | People from low socio-economic status |  |
| Older adults |  | Families |  |
| Workforce/employees |  | Indigenous people |  |
| Women |  | Migrant populations |  |
| People with disabilities |  | General population |  |
| Pregnant women |  |  |  |
| Clinical populations/chronic disease patients |  | Other (please specify): |  |

1. Please list **any international documents that have been useful** in the development of physical activity-related policy in your sector. Please rate the documents below on the scale from 1 (= “not at all useful”) to 5 (= “very useful”). Repeat as needed

| Not at all useful Very useful | | | | | | |
| --- | --- | --- | --- | --- | --- | --- |
|  | 1 | 2 | 3 | 4 | 5 | Don’t know |
| For example: the World Health Organization’s 2020 Guidelines on physical activity and sedentary behaviour: <https://www.who.int/publications-detail-redirect/9789240015128> |  |  |  |  |  |  |
| Other document (please specify): |  |  |  |  |  |  |
| Other document (please specify): |  |  |  |  |  |  |
| Other document (please specify): |  |  |  |  |  |  |
| Other document (please specify): |  |  |  |  |  |  |

1. Please use this space to provide **any further details or comments** you were not able to provide in other sections of the tool.

## Annex 2. Semi-structured key informant interview guide:

| **INTERVIEWEE NAME** | |  | | **CONDUCTED BY** | | |  | |
| --- | --- | --- | --- | --- | --- | --- | --- | --- |
| **INTERVIEW DATE** | |  | **INTERVIEW START TIME** | |  | | **INTERVIEW END TIME** |  |
|  | | | | | | | | |
| **POSITION TITLE** |  | | | **POSITION DEPARTMENT** | |  | | |
| **DESCRIPTION OF POSITION ROLES AND RESPONSIBILITIES** | | | | | | | | |
|  | | | | | | | | |

**INTRODUCTION**

- Welcome the interviewee
  - Thank you for taking the time to speak with us today. The purpose of this interview is to learn about current and planned physical activity policies and interventions in Saudi Arabia (2016-2021). It is part of a larger work led by King Faisal Specialist Hospital and Research Center (KFSHRC) and the World Bank on physical activity promotion in the Kingdom. The information you’ll provide will help us document progress and identify opportunities for improvement. The interview will take no more than one hour to complete
- Explain the interview process
  - Your participation is voluntary, and you can skip a question or stop the interview at any point. All the interviews will be confidential and will not include your name or position title. The information you provide will only be reported in aggregate with other information we collect on physical activity and sedentary behavior policies and interventions in the Kingdom.

| **Do you consent to participate in this interview?** | |
| --- | --- |
| Yes (proceed) | No (thank him or her and end the interview) |
|  |  |

- Ask for permission to record the interview
  - To ensure we capture all the conversation efficiently, we would like to ask for your permission to record it. Recordings of this conversation will be transcribed into a written document so that we may have an accurate record of the conversation for analysis. The recordings will be accessible to the researchers only and deleted after verification of the transcription.

| **Do you consent to record this interview?** | |
| --- | --- |
| Yes (proceed) | No (do the interview without recording) |
|  |  |

- Thank you very much. We will now begin the interview
  - If you need to think about a question for a little while before answering, please take as much time that you need. Do not feel like you are under any pressure to answer these questions quickly or in a certain way. There is no right or wrong answer.

**SECTION 1: Background and physical activity/sedentary behavior landscape in KSA:**

- Describe your institutions’ role in physical activity promotion or sedentary behavior reduction.
- In your opinion, which ministries/department/sectors within government are actively engaged in physical activity promotion in KSA?
  - Describe their roles
  - For example: Ministry of Health is responsible for physical activity and health policy, initiation and delivery of frameworks for action plans/programs, guidelines and subsidies

Are there other ministries/departments/sectors that are not involved currently but should/could be involved?

- What about important organizations outside government that are actively engaged in physical activity promotion?
  - This could include national sporting organizations, NGOs, charities, advocacy groups, the academic or scientific community, among others.
- In your opinion, which of these entities provides leadership for physical activity promotion in KSA?
  - **Leadership** refers to the provision of overall direction for Health-Enhancing Physical Activity (HEPA); e.g., responsibility for defining, supervising, monitoring, and managing the national physical activity agenda.
- Based on your experience, are any mechanisms in place to ensure cross-sectoral collaboration on the promotion of physical activity at a national level?
  - **Coordination** means communication on, and alignment of actions and developments relating to HEPA, and could include facilitation of regular exchange between relevant stakeholders. For example, multisectoral committee or working group.
  - If yes, briefly describe.
    - Probe for information on who is involved, who is leading these efforts, and how these collaborations function in practice.
    - Probe them to Please mention (to the extent possible) any positive or more difficult experiences. This may also include examples of collaboration with the private and voluntary sectors.
- Does any professional network or system exist in your country that links and/or supports professionals interested or currently working in physical activity or related areas?
  - If yes, please describe
- Within the past 5 years (2016-2021), how has political commitments changed in the in KSA when it comes to physical activity promotion?
  - Probe for key past events such as political changes, position statements or scientific events that have shaped the HEPA agenda.
- Does your institution have funding specifically allocated for physical activity or sedentary behavior related activities?
  - How much? Is it recurrent or permeant?

**SECTION 2: Goals, targets, and surveillance**

- Does KSA have health surveillance or monitoring system that includes measures of physical activity or sedentary behavior?
  - If yes, please provide for each population subgroup
- Was this surveillance data used to influence policy development?
  - if yes how?
- Have data on the prevalence of physical activity or sedentary behavior or other related factors influenced policy development in your sector?
  - if yes, how?
    - For example, have surveillance data been used to define national goals and targets
  - If no, why not?
    - For example, do the questions asked in the survey not provide information or the frequency not in line with policy development.
- How did you go about setting your own targets for physical activity or sedentary behavior (ministry/authority/sector)?
- How do you monitor progress towards your goals?
  - Do you have a monitoring system that includes measures of physical activity or sedentary behavior?
  - If yes, which groups are included in this monitoring system? (For example, children, elderly … etc.)
  - If not, why not?

**SECTION 3: Current and planned physical activity/sedentary behavior**

**policies and interventions by sector**

- During the development of the most important policies/action plans, was a **consultative process** used, involving relevant stakeholders?
  - If yes, please describe the process
  - Describe any challenges in engaging government ministries or other agencies through such processes.
- What mechanisms do you have in place to ensure that the key policy documents are based on the best-available scientific evidence?
  - For example, formal committees or institutions?
- Do you have a current national communication strategy (using mass media) aimed at raising awareness and promoting physical activity?
  - If yes, please provide details (e.g., posters or websites) and whether there is a brand or slogan.
- Please provide one or two examples (if any) of large-scale (preferably national) programs or interventions in each of the settings listed.
  - Please describe including name, lead organization, approach, participants, results.) and a source where further information can be obtained.
- What other policies or interventions do you have planned in the future?
- Is there any evaluation of the current physical activity promotion/ sedentary behavior reduction policies/action plans?
  - Describe

**SECTION 4: Challenges and opportunities (2016-2021):**

- What are the areas of biggest challenges faced by your sector in promotion of physical activity or reduction of sedentary behavior?
- What are the areas of greatest progress in promotion of physical activity or reduction of sedentary behavior in your sector?
- Based on your experience, what suggestions do you have to promote physical activity or reduce sedentary behavior on a national level?

**SECTION 5: Conclusion**

- Summarize the main discussion points.
- Ask the interviewee if they would like to add anything else.
- Thank them for their time.
